# Development and validation of a nomogram for predicting sepsis in patients with pyogenic liver abscess

**DOI:** 10.1038/s41598-023-37907-2

**Published:** 2023-07-05

**Authors:** Ji Li, Yin Wang, Jinhong Luo, Zhikun Yin, Weifeng Huang, Jinyan Zhang

**Affiliations:** 1 Department of Gastroenterology and Hepatology, The First Affiliated Hospital of Xiamen University, School of Medicine, Xiamen University, Xiamen, 361003 China; 2 Department of Gastroenterology, The Third Hospital of Xiamen, Xiamen, China

**Keywords:** Diseases, Gastroenterology, Medical research, Risk factors

## Abstract

Pyogenic liver abscess (PLA) is a severe condition that significantly increases the risk of sepsis. However, there is a notable dearth of research regarding the prediction of sepsis in PLA patients. The objective of this study was to develop and validate a prognostic nomogram for predicting sepsis in PLA patients. A total of 206 PLA patients were enrolled in our study, out of which 60 individuals (29.1%) met the Sepsis-3 criteria. Independent risk factors for sepsis were identified through univariate and multivariate logistic regression analyses. Subsequently, a nomogram was developed based on age, positive blood culture, procalcitonin, alanine aminotransferase, blood urea nitrogen, and d-dimer. The nomogram demonstrated excellent calibration and discrimination, as evidenced by the area under the receiver operating characteristic curve (AUC) values of 0.946 (95% confidence interval [CI], 0.912–0.979) and 0.980 (95%CI 0.951–1.000) in the derivation and validation cohorts, respectively. Furthermore, decision-curve analysis confirmed the clinical utility of the nomogram. This study provides valuable insights for the prevention of sepsis in PLA patients and underscores the potential application of the prognostic nomogram in clinical practice.

## Introduction

Pyogenic liver abscess (PLA) is an infectious disease that presents a significant global public health concern, particularly in developing countries where its incidence rate has been increasing^1,2^. Despite advancements in our understanding of the underlying pathogens, diagnostic techniques, and therapeutic approaches, PLA remains associated with severe complications, resulting in fatality rates ranging from 7.8 to 28.6%^3^. Sepsis, a grave complication of PLA, can lead to multi-organ dysfunction, septic shock, and death^4,5^. The prognosis for patients with PLA who develop sepsis is generally unfavorable^2,6,7^. However, accurately predicting the prognosis of sepsis proves challenging due to its inherent heterogeneity^4,8^. Consequently, the identification of effective strategies for sepsis prevention and risk management becomes crucial.

Nomograms are visual tools that consider relevant risk factors to predict the probability of a clinical event^9,10^. However, there is a lack of research on identifying risk factors and developing prognostic models for sepsis in PLA patients. Therefore, this study aimed to analyze clinical data from PLA patients and develop a nomogram to accurately predict the probability of sepsis. The resulting tool will serve as a valuable resource for clinicians, aiding in the prevention and management of sepsis in PLA patients.

## Results

### Clinical characteristics of patients

**Figure 1:**
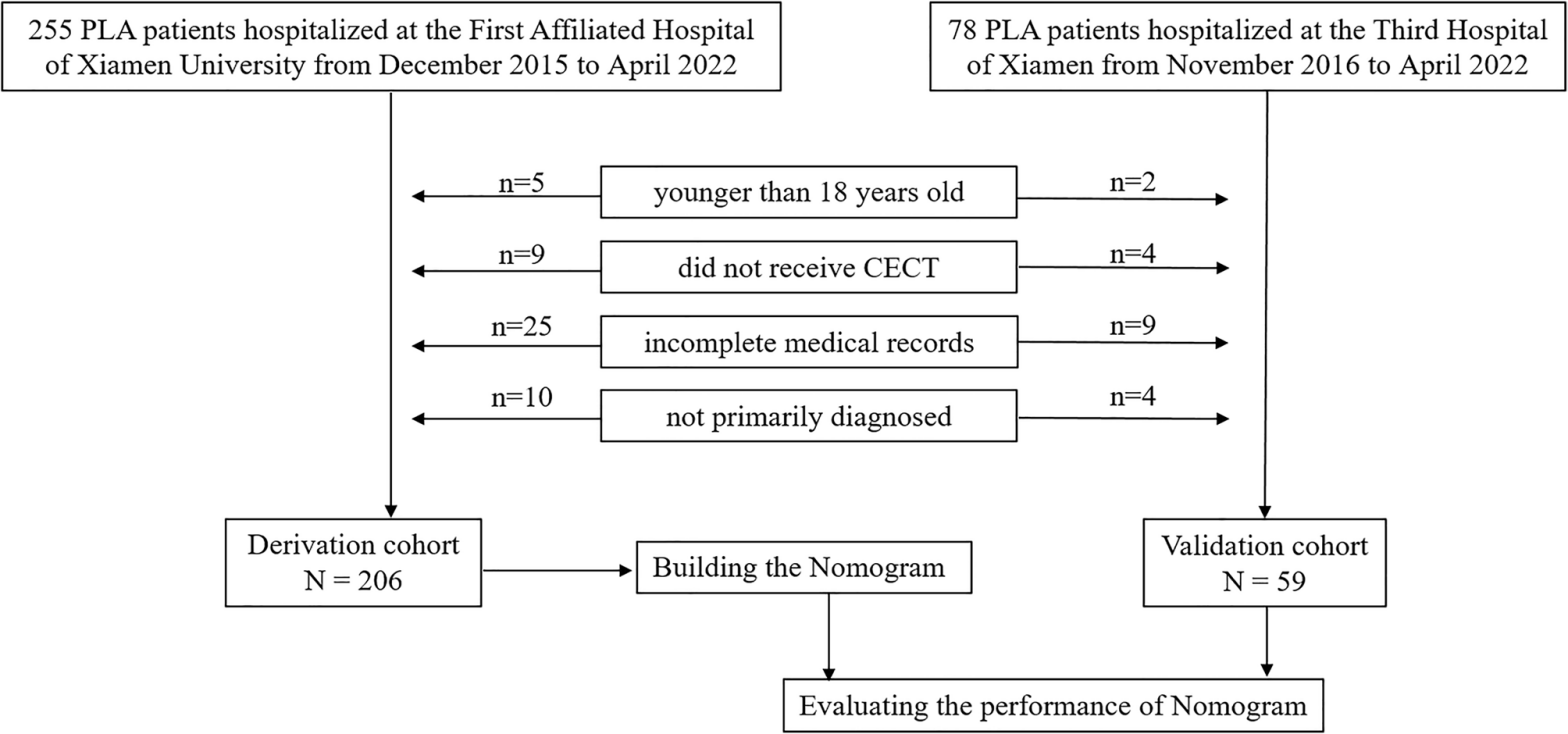
Flowchart of the study design. CECT, contrast-enhanced computerized tomography.

The study included 206 patients in the derivation cohort from the First Affiliated Hospital of Xiamen University and 59 patients in the validation cohort from the Third Hospital of Xiamen (Fig. 1). In the derivation cohort, 60 out of 206 (29.1%) patients were diagnosed with sepsis, while in the validation cohort, 19 out of 59 (32.2%) patients had sepsis. There was no significant difference in the prevalence of sepsis between the two cohorts (*P* = 0.649).

A comparison of demographic and clinical characteristics, vital signs, laboratory findings, radiological findings, and clinical outcomes was conducted between PLA patients with and without sepsis in the derivation cohort, as summarized in Table 1. The analysis revealed that age, blood culture, laboratory findings including C-reactive protein, procalcitonin, alanine aminotransferase (ALT), aspartate aminotransferase (AST), albumin, total bilirubin, blood urea nitrogen (BUN), creatinine, lymphocyte, neutrophil-to-lymphocyte ratio (NLR), platelet, prothrombin time, and d-dimer, as well as radiological findings such as septations within the abscess, rim enhancement, internal gas bubble, and pleural effusion were significantly associated with sepsis in the derivation cohort. Furthermore, patients with sepsis were more likely to suffer from septic shock, require intensive care unit admission, and experience infection-related deaths.

**Table 1:** Comparison of clinical data between patients with and without sepsis in the derivation cohort.

Variables	Sepsis (+)	Sepsis (−)	*P* value
N	60	146	
Age	61.00 (56.00–68.00)	57.00 (49.00,66.00)	0.016
Sex, male (%)	42 (70.0)	101 (69.2)	0.907
Pulse rate (beats/min)			0.756
70–109, n (%)	47 (78.3)	117 (80.1)	
110–139 or 55–69, n (%)	13 (21.7)	28 (19.1)	
140–179, n (%)	0 (0.0)	1 (0.7)	
Mean arterial pressure (mmHg)			
70–109, n (%)	51 (85.0)	126 (86.3)	0.807
110–129 or 50–69, n (%)	9 (15.0)	20 (13.7)	
Respiratory rate (breaths/min)			
12–24, n (%)	5 (8.3)	10 (6.8)	0.762
25–34, n (%)	55 (91.7)	135 (92.5)	
35–49, n (%)	0 (0.0)	1 (0.7)	
Glasgow Coma Scale			
15, n (%)	4 (6.7)	13 (8.9)	0.782
13–14, n (%)	56 (93.3)	133 (91.1)	
Symptoms			
Fever (%)	54 (90.0)	133 (91.1)	0.805
Abdominal pain (%)	20 (33.3)	61 (41.8)	0.259
Gastrointestinal symptoms (%)	19 (31.7)	47 (32.2)	0.942
Comorbidities			
Diabetes mellitus (%)	30 (50.0)	59 (40.4)	0.207
Hypertension (%)	18 (30.0)	34 (23.3)	0.314
Biliary disorder (%)	15 (25.0)	34 (23.3)	0.793
Malignancy (%)	7 (11.7)	11 (7.5)	0.340
Smoking (%)	15 (25.0)	32 (21.9)	0.632
Drinking (%)	11 (18.3)	33 (22.6)	0.497
Treatment			
Antibiotics only (%)	29 (48.3)	65 (44.5)	0.618
Antibiotics + abscess drainage/surgery (%)	31 (51.7)	81 (55.5)	
Pathogen in blood			
Negative (%)	16 (26.7)	119 (81.5)	< 0.001
Positive (%)	44 (73.3)	27 (18.5)	
Pathogen in pus			
Negative (%)	36 (60.0)	89 (61.0)	0.898
Positive (%)	24 (40.0)	57 (39.0)	
Laboratory findings			
C-reactive protein (mg/L)	144.99 (90.73, 191.64)	97.13 (63.8, 158.19)	< 0.001
Procalcitonin (ng/mL)	45.11 (13.46, 99.35)	1.62 (0.55, 11.47)	< 0.001
AST (U/L)	102.50 (54.50, 190.25)	39.00 (24.00, 64.00)	< 0.001
ALT (U/L)	97.75 (48.00, 174.25)	58.00 (32.75, 90.00)	< 0.001
GGT (U/L)	101.50 (53.25, 186.50)	125.50 (61.50, 215.00)	0.142
Alkaline phosphatase (U/L)	149.50 (89.50, 223.75)	148.00 (105.00, 203.00)	0.989
Albumin (g/L)	29.25 (26.55, 32.63)	32.25 (28.40, 35.70)	< 0.001
Total bilirubin (μmol/L)	28.40 (16.83, 44.68)	14.65 (9.78, 23.63)	< 0.001
Creatinine (μmol/L)	98.00 (68.50, 131.25)	63.50 (51.75, 80.25)	< 0.001
BUN (mmol/L)	8.91 (6.21, 14.97)	4.77 (3.60, 6.30)	< 0.001
White blood cell (10^9^/L)	12.81 (9.09, 18.82)	12.90 (9.71, 15.62)	0.688
Neutrophil (10^9^/L)	11.71 (8.05, 17.19)	10.31 (7.83, 13.14)	0.088
Lymphocyte (10^9^/L)	0.56 (0.40, 0.80)	1.11 (0.73, 1.53)	< 0.001
NLR	21.38 (13.38, 33.43)	9.09 (5.79, 15.50)	< 0.001
Hemoglobin (g/L)	117.50 (104.25, 129.75)	121.00 (110.75, 133.00)	0.108
Platelet (10^9^/L)	87.00 (40.00, 153.00)	243.00 (162.75, 342.75)	< 0.001
PLR	158.50 (81.56, 330.30)	211.89 (148.60, 337.40)	0.006
Prothrombin time (s)	15.30 (13.63, 16.50)	14.00 (13.10, 15.10)	< 0.001
APTT (s)	37.55 (31.88, 41.13)	38.20 (28.40, 45.60)	0.702
d-dimer (mg/L)	5.74 (2.52, 13.56)	2.05 (1.20, 3.48)	< 0.001
Radiologic findings			
Abscess diameter (cm)	6.30 (4.60, 8.18)	6.45 (4.40, 8.95)	0.987
Abscess number, single (%)	53 (88.3)	112 (76.7)	0.058
Location			
Left (%)	9 (15.0)	43 (29.5)	0.060
Right (%)	45 (75.0)	85 (58.2)	
Both (%)	6 (10.0)	18 (12.3)	
Septations within the abscess, unilocular (%)	37 (61.7)	111 (76.0)	0.037
Rim enhancement (%)	25 (41.7)	91 (62.3)	0.007
Septal enhancement (%)	23 (38.3)	57 (39.0)	0.925
Necrotic debris (%)	5 (8.3)	16 (11.0)	0.571
Internal gas bubble (%)	16 (26.7)	13 (8.9)	0.001
Biliary gas (%)	6 (10.0)	7 (4.8)	0.280
Pylephlebitis (%)	0 (0.0)	1 (0.7)	1.000
Pleural effusion (%)	45 (75.0)	66 (45.2)	< 0.001
Spontaneous rupture (%)	2 (3.3)	2 (1.4)	0.710
Clinical outcomes			
Septic shock (%)	42 (70.0)	3 (2.1)	< 0.001
Intensive care unit admission (%)	50 (83.3)	12 (8.2)	< 0.001
Related death (%)	3 (5.0)	0 (0.0)	0.024

AST, aspartate aminotransferase; ALT, alanine aminotransferase; GGT, gamma-glutamyltransferase; BUN, blood urea nitrogen; NLR, Neutrophil-to-lymphocyte ratio; PLR, Platelet-to-lymphocyte ratio; APTT, activated partial thromboplastin time.

### Development of a novel nomogram prediction model

**Figure 2:**
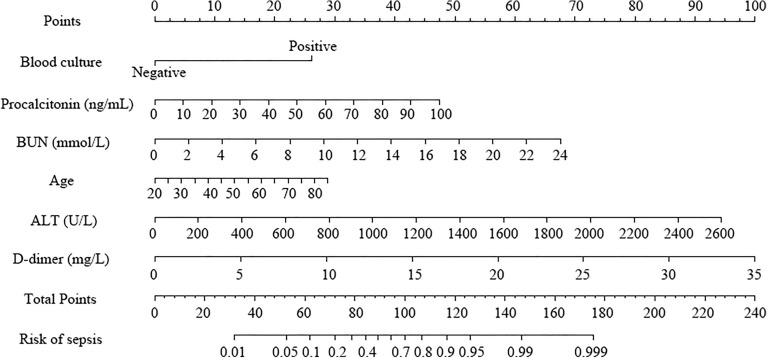
Nomogram for prediction of sepsis in pyogenic liver abscess. ALT, alanine aminotransferase; BUN, blood urea nitrogen.

**Figure 3:**
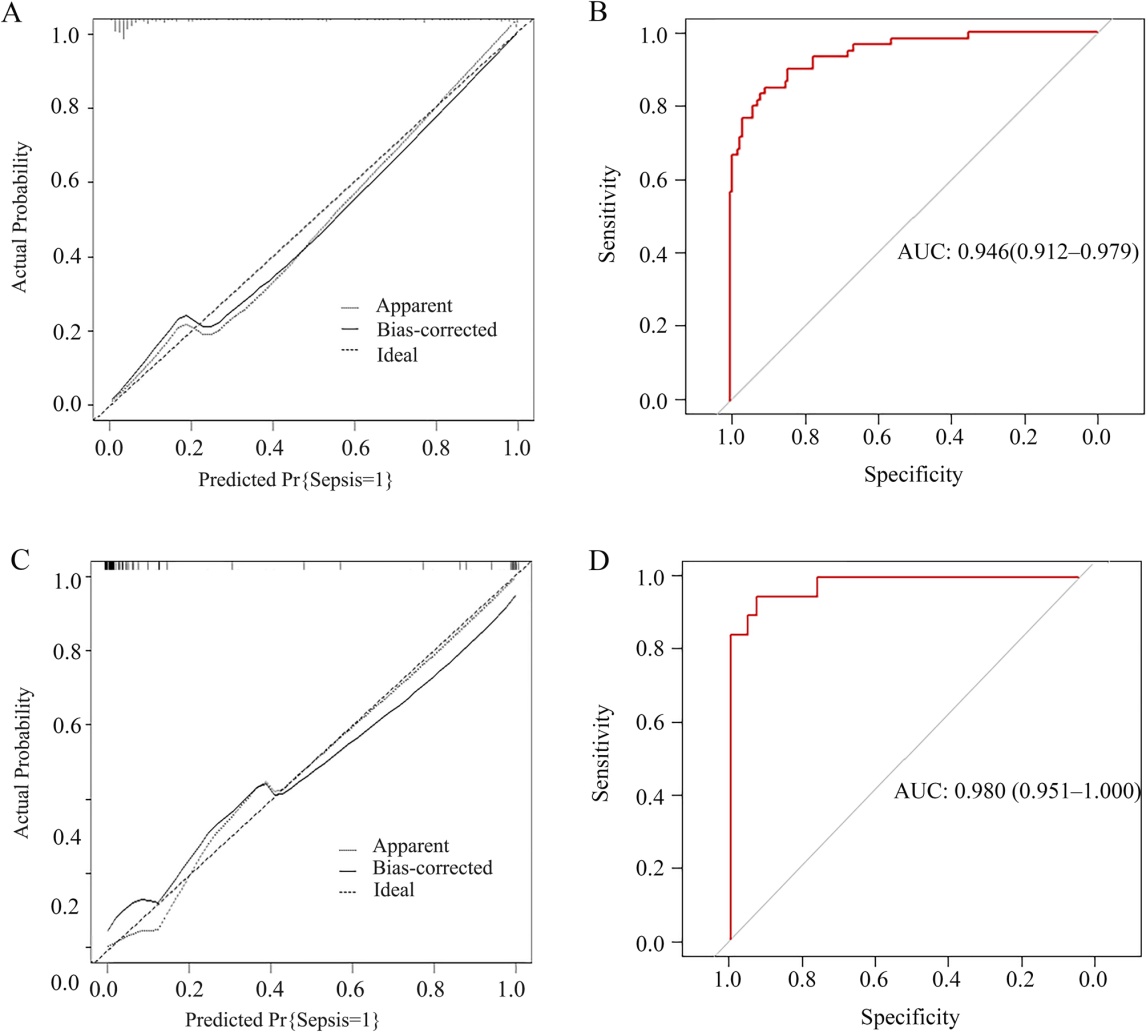
Calibration and discrimination curves of the prognostic nomogram model. Calibration curves (**A**) and discrimination curve (**B**) of the derivation cohort. Calibration curves (**C**) and discrimination curve (**D**) of the validation cohort. ROC, receiver operating characteristic; AUC, area under ROC curve.

Multivariate logistic regression analysis revealed age, positive blood culture, procalcitonin, ALT, BUN, and d-dimer were independent risk predictors of sepsis in PLA patients within the derivation cohort (Table 2). A nomogram was then developed to integrate these predictors, and the risk score of sepsis was calculated by summing their respective scores using R software (Fig. 2). The calibration curve of the nomogram demonstrated a high degree of concordance between the predicted and observed sepsis outcomes (Fig. 3A). Furthermore, the Hosmer–Lemeshow test yielded a non-significant *P* value of 0.939, coupled with a brier score of 0.077, indicating excellent calibration accuracy. The nomogram also exhibited strong predictive capability for sepsis, as evidenced by an area under the receiver operating characteristic (ROC) curve (AUC) of 0.946 (95% confidence interval [CI], 0.912–0.979) (Fig. 3B).

**Table 2:** Univariate and multivariate logistics regression analysis of sepsis in the derivation cohort.

	Univariable OR (95%CI)	*P* value	Multivariable OR (95%CI)	*P* value
Age	1.035 (1.008–1.062)	0.012	1.074 (1.010–1.143)	0.023
Positive blood culture	12.120 (5.968–24.615)	< 0.001	7.461 (1.882–29.581)	0.004
C-reactive protein	1.007 (1.002–1.011)	0.002	0.996 (0.984–1.008)	0.546
Procalcitonin	1.050 (1.034–1.066)	< 0.001	1.065 (1.032–1.098)	< 0.001
AST	1.009 (1.004–1.013)	< 0.001	0.992 (0.984–1.001)	0.073
ALT	1.010 (1.005–1.014)	< 0.001	1.011 (1.001–1.020)	0.030
Albumin	0.884 (0.829–0.943)	< 0.001	0.896 (0.775–1.035)	0.137
BUN	1.408 (1.260–1.573)	< 0.001	1.227 (1.040–1.448)	0.015
Lymphocyte	0.088 (0.036–0.214)	< 0.001	0.352 (0.065–1.894)	0.224
NLR	1.075 (1.045–1.107)	< 0.001	1.022 (0.981–1.066)	0.297
d-dimer	1.337 (1.202–1.487)	< 0.001	1.207 (1.013–1.439)	0.036
Prothrombin time	1.486 (1.233–1.792)	< 0.001	0.879 (0.595–1.297)	0.515
Septations within the abscess, Unilocular	1.971 (1.035–3.755)	0.039	1.970 (0.530–7.323)	0.311
Rim enhancement	0.432 (0.234–0.797)	0.007	0.348 (0.091–1.330)	0.123
Internal gas bubble	3.720 (1.659–8.341)	0.001	3.197 (0.472–21.634)	0.233
Pleural effusion	3.636 (1.862–7.100)	< 0.001	3.555 (0.868–14.557)	0.078

AST, aspartate aminotransferase; ALT, alanine aminotransferase; BUN, blood urea nitrogen; NLR, Neutrophil-to-lymphocyte ratio.

### Validation of the nomogram prediction model

In the validation cohort, we found that our nomogram model was well calibrated (Fig. 3C), with a brier score of 0.047 and a non-significant *P* value of 0.953 in the Hosmer–Lemeshow test. Additionally, the nomogram exhibited excellent discrimination for sepsis prediction, with an AUC of 0.980 (95%CI 0.951–1.000) (Fig. 3D).

### Decision curve analysis

**Figure 4:**
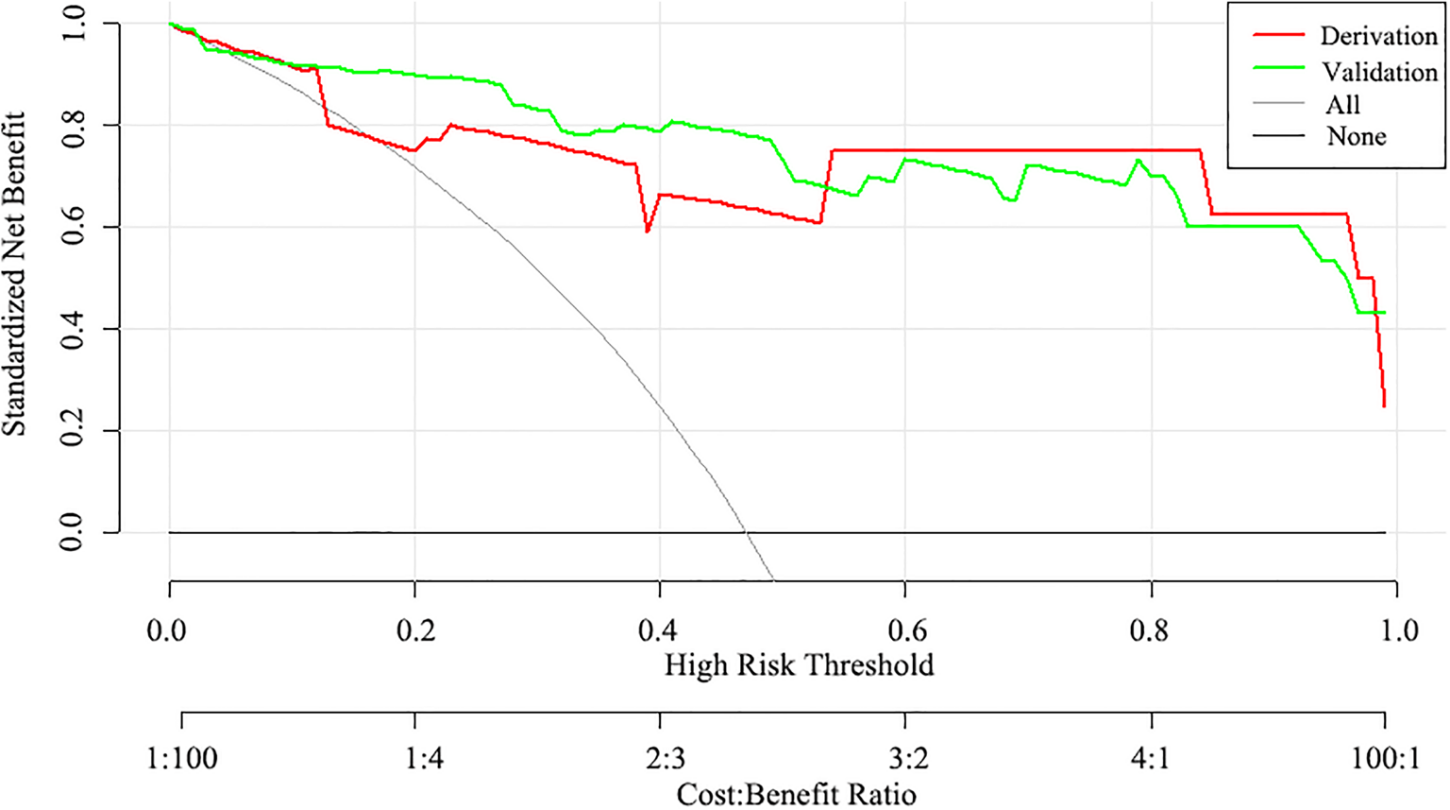
The decision curve analysis curves of the nomogram model in the derivation (red line) and validation (green line) cohort.

To evaluate the clinical utility of our nomogram model, we conducted a decision curve analysis to calculate the net benefits across a range of threshold probabilities (Fig. 4). The results demonstrated that our model was a reliable tool for predicting sepsis in patients with PLA, as it achieved good clinical application across nearly the entire range of threshold probabilities.

## Discussion

Patients diagnosed with PLA who subsequently develop sepsis are known to face an elevated risk of encountering clinical complications and unfavorable prognoses^2^. It is widely recognized that early and precise risk assessment, along with the administration of appropriate antibiotics and targeted treatment, are critical for successfully managing sepsis in these individuals^11^. Consequently, the identification of risk factors associated with sepsis in PLA patients assumes paramount importance, as it has the potential to significantly enhance clinical outcomes and reduce the incidence of sepsis.

The primary objective of our study was to develop and validate a nomogram model to predict the probability of sepsis in PLA patients during hospitalization. As total bilirubin, creatinine, and platelet levels are already included in the definition of Sepsis-3, we did not incorporate these parameters into our model. Through our analysis, we identified age, positive blood culture, procalcitonin, ALT, BUN, and d-dimer as the most optimal predictors of sepsis in PLA patients. Importantly, our nomogram model exhibited excellent calibration and discrimination capabilities in both derivation and external validation cohorts. Furthermore, the decision curve analysis provided additional insights into the clinical utility of our nomogram model. The analysis demonstrated its potential to guide disease management and treatment planning by generating favorable net benefits across various threshold probabilities. Overall, our findings suggest that our nomogram model could serve as a valuable tool for clinicians to predict the probability of sepsis in PLA patients and guide early intervention to improve patient outcomes.

Advanced age is a crucial factor that significantly affects the prognosis and clinical severity of numerous illnesses^12^. However, its influence on PLA remains largely unknown. Studies investigating the role of age in complications of PLA, such as sepsis and septic shock, in the geriatric population have reported controversial findings^12–15^. In our study, we found that age was an independent risk factor for sepsis in PLA patients. Elderly patients with PLA may present with atypical and misleading symptoms and signs upon admission, which can result in the severity of the illness being underestimated^14^. Moreover, elderly patients with PLA often have multiple chronic comorbidities, which could lead to the progression of the disease and the occurrence of sepsis. Our findings suggest that age should be considered an important risk factor in the early identification and management of sepsis in PLA patients, particularly in the elderly population.

Bacterial infection via the biliary tract or organs drained by portal veins is a well-established contributor to the development of PLA, which frequently results in sepsis and organ dysfunction. Currently, there are limited studies comparing clinical outcomes between culture-positive and culture-negative PLA patients. One study reported no difference in the incidence of sepsis between culture-negative and culture-positive liver abscess patients when blood and pus cultures were both considered^16^. In contrast, our study revealed an increased risk of sepsis in PLA patients with a positive blood culture alone. For the treatment of PLA, abscess drainage and administration of appropriate antibiotics are the primary therapeutic modalities^17,18^. The choice of antibiotic should be based on the suspected source of infection and the predominant pathogens in the region. It is worth noting that a positive blood culture in PLA patients indicates not only the presence of bacteria in the bloodstream but also an increased risk of sepsis. Therefore, empirical antibiotic therapy should target all potential pathogens, using potent antibiotics such as carbapenems, and may require an extended duration. Culture and susceptibility testing should guide antibiotic therapy, and patients should be closely monitored to ensure treatment efficacy. Prompt attention should be given to any signs of sepsis, warranting immediate intervention.

Procalcitonin is a widely used biomarker for sepsis, providing a real-time response that increases transiently during sepsis and is long enough to detect^19^. Compared to other biomarkers such as C-reactive protein, necrosis factor-alpha, and interleukins, procalcitonin has been shown to be more sensitive and specific in sepsis diagnosis^20,21^. In particular, procalcitonin has been identified as a marker for sepsis diagnosis in liver abscess caused by transcatheter arterial chemoembolization (TACE) therapy among patients with hepatocellular carcinoma^22^. Our study further demonstrated the strong predictive value of procalcitonin in patients with sepsis due to pyogenic liver abscess. Therefore, we recommend the routine procalcitonin examination as an early screening tool for sepsis in these patients to prompt timely and effective treatment.

d-dimer, a specific degradation product of stabilized fibrin, serves as a valuable biomarker for assessing the hypercoagulable state and secondary hyperfibrinolysis^23^. Within the context of sepsis, a complex interplay of pathophysiological processes occurs, involving the release of inflammatory mediators, endothelial cells, and cytokines. These elements collectively activate and amplify the coagulation cascade, leading to a substantial increase in d-dimer concentrations and other coagulation-related biomarkers. Previous studies have already established a correlation between d-dimer levels and the severity of sepsis, as well as their predictive value in determining in-hospital sepsis mortality^24,25^. In our comprehensive analysis of patients with PLA, we substantiates that the concentration of d-dimer upon admission holds potential as a reliable predictor for sepsis in PLA patients. By recognizing the role of d-dimer as a predictive marker, physicians can enhance their ability to promptly identify sepsis, thereby leading to improved patient outcomes. Further research is warranted in this area to elucidate the precise mechanisms linking d-dimer and sepsis, thereby facilitating the development of targeted interventions to combat this life-threatening condition.

Kidney and liver dysfunction represent significant pathological manifestations induced by infection. BUN, the primary end product of protein metabolism, is predominantly excreted by the kidneys in the human body. BUN levels serve as an indicator of protein catabolism and can also reflect renal impairment^26^. Patients with sepsis often experience a substantial increase in the rate of protein catabolism, which is frequently accompanied by acute renal injury^27^. As a result, BUN levels tend to rise in septic patients. Aminotransferases, such as ALT, are commonly used to assess hepatocellular damage. Elevated ALT levels in clinical practice are indicative of acute hepatocellular injury^24^. Our study demonstrates the predictive value of BUN and ALT in assessing sepsis among patients with PLA. Given that sepsis-induced liver and kidney dysfunction can manifest subtly, it is crucial to consider the possibility of such dysfunction in any patient presenting with an infection, particularly when ALT and BUN levels are elevated.

However, our study has certain limitations that should be considered. Firstly, its retrospective nature and reliance on electronic medical record systems may introduce potential biases. Furthermore, certain important parameters such as partial pressure of oxygen and serum lactate level were not included in our study due to their infrequent evaluation at admission in our hospital. Future studies should investigate the potential impact of these variables on the prediction of sepsis. Secondly, the predictive value of blood cultures may be constrained by the time required to confirm a positive result. Thirdly, while we conducted external validation using an additional database from another hospital, both databases were obtained from the same region, which may result in some regional deviations and limit generalizability. Lastly, although our study included a relatively large derivation cohort of 206 patients, and a total of 265 patients which exceeds the sample size of many previous studies, further validation using larger databases from prospective studies conducted in different regions or countries is warranted to strengthen the robustness of our findings.

In conclusion, we have developed a novel nomogram that incorporates age, positive blood culture, procalcitonin, ALT, BUN, and d-dimer to predict the probability of sepsis in patients with PLA. Our nomogram is easily accessible, cost-efficient and has demonstrated good calibration, discrimination, and clinical utility. The results of our study have important significance for clinical practice, as the nomogram may assist clinicians in predicting sepsis occurrence in PLA patients and guiding clinical management. Overall, our study provides a valuable tool for predicting sepsis in PLA patients and highlights the importance of early detection and treatment.

## Methods

### Patients

A retrospective study was conducted at the First Affiliated Hospital of Xiamen University between December 2015 and April 2022 to develop a prediction model for PLA. A validation dataset was also collected between November 2016 and April 2022 at the Third Hospital of Xiamen. The study recruited hospitalized adult patients who were diagnosed with pyogenic liver abscess based on clinical presentation, abdominal imaging findings, positive bacterial cultures of blood or pus, and exclusion of alternative diagnoses such as amoebic or tuberculous liver abscess^3,28^. Inclusion criteria were as follows: (1) all patients diagnosed with PLA, (2) older than 18 years old, and (3) presence of a liver focal lesion or multiple lesions on contrast-enhanced computerized tomography (CECT) images with characteristics compatible with pyogenic liver abscess. Exclusion criteria included patients who were not primarily diagnosed with PLA, and those who had incomplete medical record**s.** The study was approved by the ethics committee of Institutional Review Boards of the First Affiliated Hospital of Xiamen University and the Third Hospital of Xiamen. The study was conducted in accordance with the Declaration of Helsinki. The Institutional Review Boards of the First Affiliated Hospital of Xiamen University and the Third Hospital of Xiamen allowed to waive the informed consent due to the retrospective nature of this study.

### Clinical data collection

Clinical data, including demographic characteristics (age and sex), vital signs (pulse rate and respiratory rate), clinical symptoms, comorbidities, treatment methods, laboratory findings, and clinical outcomes were collected from the electronic medical record systems of the hospitals. All patients with PLA underwent CECT before drainage of the liver abscess. CECT scans were reviewed for the purpose by two radiologists, and any disagreement was resolved by a third party. The features were collected as previously described^29–31^.

The assessment of sepsis was conducted within the first 72 h of hospitalization and continued throughout the entire duration of the patient's hospital stay. Sepsis was defined as life-threatening organ dysfunction caused by infection with Sequential Organ Failure Assessment score (SOFA) ≥ 2, according to the Third International Consensus Definitions for sepsis and septic shock (Sepsis-3) updated in 2016^4^.

### Statistical analysis

The chi-squared test or Fisher’s exact test was used to analyze categorical variables, and nonparametric tests were used to analyze continuous variables. Continuous variables were described by median (interquartile range [IQR]), and categorical variables were expressed as frequency rates and percentages.

Univariate analysis was performed to identify potential predictors with a significance level of *P* < 0.05, which were then included in multivariate logistic regression. Variables with *P* < 0.05 in the multivariable analysis were used to develop a nomogram in the derivation cohort. Calibration curves with 1000 bootstrap samples were plotted to demonstrate the consistency between the predicted probability and the actual probability. The ROC curve was used to evaluate the diagnostic efficacy of the nomogram. The discriminative ability of the nomograms was further measured by AUC. Additionally, the nomogram was validated in an external cohort, and its clinical utility was assessed by decision-curve analysis through calculating net benefits at different threshold probabilities. R version 4.2.0 (Math Soft, Cambridge, Massachusetts) was used for all statistical analyses, and *P* < 0.05 (two-tailed) was considered statistically significant.

## Data Availability

The data supporting the findings of this study are available from the first author upon request. The data are not publicly available due to privacy or ethical restrictions.
